# Microarrays, deep sequencing and the true measure of the transcriptome

**DOI:** 10.1186/1741-7007-9-34

**Published:** 2011-05-31

**Authors:** John H Malone, Brian Oliver

**Affiliations:** 1 Laboratory of Cellular and Developmental Biology, National Institute of Digestive, Diabetes, and Kidney Diseases, National Institutes of Health, Bethesda, MD 20892, USA

## Abstract

Microarrays first made the analysis of the transcriptome possible, and have produced much important information. Today, however, researchers are increasingly turning to direct high-throughput sequencing - RNA-Seq - which has considerable advantages for examining transcriptome fine structure - for example in the detection of allele-specific expression and splice junctions. In this article, we discuss the relative merits of the two techniques, the inherent biases in each, and whether all of the vast body of array work needs to be revisited using the newer technology. We conclude that microarrays remain useful and accurate tools for measuring expression levels, and RNA-Seq complements and extends microarray measurements.

## Transcriptional profiling

The transcriptome, the entire repertoire of transcripts in a species, represents a key link between information encoded in DNA and phenotype. A fully quantitatively described transcriptome is dauntingly large. For example, there are more than 3 billion bases in the human genome, about 10^14 ^cells in the body, each cell has about 300,000 molecules of RNA [1], and the average gene size is about 28 kilobase pairs [2]. Thus, for a full representation of a human, there are about 8.4^23 ^(280000 × 300000 × 10^14^) RNA bases in the full transcriptome. The tools for profiling RNA have been available for years, as Northern blots, reverse-transcription PCR (RT-PCR), expressed sequence tags (ESTs), and serial analysis of gene expression (SAGE). But the rapid and high-throughput quantification of the transcriptome became a possibility only with the development of gene expression microarrays [3]. With the more recent advent of techniques for direct sequencing of the transcriptional output of the genome, we can now at least begin to think about a complete transcriptional characterization of all the cells of an organism.

## Microarrays

Gene expression microarray results have produced much important information about how the transcriptome is deployed in different cell types [4] and tissues [5], how gene expression changes across development states [6,7] and disease phenotypes [8,9], and how it varies within [10] and between species [11]. They have also led to surprising and contentious conclusions on how much of the genome is transcribed into non-coding RNAs.

The starting point for a microarray is a set of short oligonucleotide probes representing genomic DNA. A typical modern microarray consists of patches of such probes complementary to the transcripts whose presence is to be investigated, and immobilized on a solid substrate. In modern arrays, probe design is usually based on genome sequence or on known or predicted open reading frames and usually multiple probes are designed per gene model. Transcripts are extracted from samples of the cell or tissues to be investigated, labeled with fluorescent dyes (either one color or two), hybridized to the arrays, washed, and scanned with a laser. Probes that correspond to transcribed RNA hybridize to their complementary target. Because transcripts are labeled with fluorescent dyes, light intensity can be used as a measure of gene expression.

Expression profiling by microarrays has been very successful. Searching the term 'microarray' in PubMed produces more than 40,000 citations. The Gene Expression Omnibus (GEO), the repository of transcriptome datasets managed by the National Center for Biotechnology Information, has more than 520,000 individual experiments archived and around 21,000 project submissions, most produced from microarrays. This impressive body of work has produced a range of mature strategies for data analysis and experimental design [12].

As we have learned more about the design, chemistry and kinetics of array assays, the quality of microarray data has improved dramatically. In the early days, microarrays designed by different companies appeared to produce different results with the same samples [13]. The fluorescent readout of hybridization intensities varied between different laser scanners and there was variation in reproducibility between different labs [14]. Ozone differentially degraded the fluorescent dyes [15]. Recognition of biases and other artifacts by individual labs and organizations, such as the MicroArray Quality Control (MAQC) consortium, has led to the development of quality control standards that operate to ensure the utility of a well performed microarray experiment [16]. For example, experimental and computational methods have been developed for dealing with systematic variation between laboratories [12,17-19]. As with any measurement tool, it is important to know the biases inherent in the technique. For microarrays, it has taken a decade to understand these biases but for microarrays this has now been achieved and stable analytical solutions have been developed.

## Deep sequencing

Meanwhile, a revolution in the analysis of RNA has come about through the development of tools for massively parallel sequencing of DNA molecules. Not very many years ago a graduate student using a slab gel electrophoresis instrument with fluorescent terminator chemistry would be excited to get 500-800 base pairs of high quality sequence data from a single gene after about a week's worth of work. For perspective, *Drosophila melanogaster *has 120 million bases in its small and compact genome and so a hard-working graduate student would need more than 400 years to complete one genome. In early genome projects, even with an entire team of people spread across both academic and commercial sectors of science it took several years of work to complete the *D. melanogaster *genome [20]. Today, roughly 10 years later, we have instruments that can sequence multiple fly genomes in a few days to a week [21]. This technology allows a DNA fragment to be repeatedly sequenced in a very short time - a procedure that is known as deep sequencing and delivers greatly increased sensitivity and accuracy. These techniques have most recently been extended to the analysis of the transcriptome by what is known as RNA-Seq [22-28]. Deep sequencing of RNA on Illumina's Genome Analyzer and HiSeq instruments as well as Applied Biosystems' SOLiD instrument are now fast-developing alternatives for profiling the transcriptome.

Instead of using molecular hybridization to 'capture' transcript molecules of interest, RNA-Seq samples transcripts present in the starting material by direct sequencing. Transcript sequences are then mapped back to a reference genome. Reads that map back to the reference are then counted to assess the level of gene expression, the number of mapped reads being the measure of expression level for that gene or genomic region.

There are several things that sequencing RNA can do that microarrays cannot. Because RNA-Seq provides direct access to the sequence, junctions between exons can be assayed without prior knowledge of the gene structure, RNA editing events can be detected, and knowledge of polymorphisms can provide direct measurements of allele-specific expression. Because microarray probes are designed on the basis of inferences from prior genomic sequence data, and light intensity is used as surrogate of gene expression, microarrays will miss exon junctions for novel expressed regions and RNA editing events, and cannot easily detect allele-specific differences in gene expression. Finally, because RNA-Seq provides direct access to the sequence this technique can be used on species for which a full genome sequence is not available, whereas the only option in this case for microarrays is to hybridize RNA to a microarray designed for another species, which has limitations because of sequence divergence.

There are also several general problems with measuring gene expression levels genome-wide that sequencing RNA might make easier. Expressed regions of the genome that correspond to genes not currently identified might be easier to detect with sequencing than with microarrays, because detection depends only on where reads map in the genome and not on whether that region is annotated. That limitation of microarrays can, however, be overcome by what are known as tiling arrays, in which overlapping probes are designed to assay sequences over the entire genome [29-32]. Tiling arrays were the basis for the discovery of genomic 'dark matter' - extensive transcription from non-coding regions of the genome. However it is difficult using tiling arrays to balance the design of probes to achieve full genome coverage while avoiding as far as possible cross-hybridization potential, and this has led to controversy about the extent of the non-coding transcriptome. RNA-Seq does not depend upon hybridization and thus does not suffer from this potential artifact.

Another strength of RNA-Seq is in the quantification of individual transcript isoforms [33,34]. Alternative splicing, the mechanism whereby different isoforms of proteins are generated, is acknowledged to be an important source of functional diversity in eukaryotes, but it has been relatively little studied at the level of the transcriptome, principally because of the difficulty of measuring expression for each isoform. Splicing arrays exist but they require probes designed to be complementary to junctions, and these can therefore be generated only if the genes and the distinct isoforms produced from them are already known [35]. Sequencing by contrast provides direct access to reads that span exon/exon boundaries and in theory makes it possible to study the expression of different isoforms for a gene and to make comparisons of isoform diversity and abundance. Additionally, sequencing appears to be better at detecting exon/exon junctions than arrays [29].

## Practical advantages and drawbacks of microarrays and RNA sequencing

So far, we have discussed the advantages and disadvantages of sequencing and arrays that are inherent in the two techniques. But there are also major practical considerations. The greatest current advantage of arrays is their relatively low cost compared with sequencing (in our lab about 10X). Presently, using a 12-plex array from Nimblegen our array costs are less than $100 per sample whereas sequencing is around $1,000 per sample. These costs will decrease as sequencing output increases. Another advantage is knowledge of biases in array data and mature analysis strategies and experimental designs for dealing with them. By comparison, sources of bias in sequence data are still being actively researched, and optimum analytical strategies developed [36]. Meanwhile RNA-Seq continues to evolve, so it will take some time to develop appropriate standards for this tool.

One of the most important concerns about sequencing RNA is the depth of sequencing required to effectively sample the transcriptome. This equates to how many times to sequence a sample. For highly expressed genes, small amounts of sequencing are sufficient, but for the middle and low end of expression levels, it is clear that many reads are needed. In the fly modENCODE samples for example, even after 50 million mapped reads new transcript discovery did not saturate [37]. In our hands, we estimate that 6-8 million mapped reads provide adequate coverage to accurately estimate roughly 80-90% of the head transcriptome in flies. Other tissues are different and this is particularly the case for genes with low levels of expression. The gene *doublesex*, a transcription factor involved in sexual dimorphism in flies, is not detected by RNA-Seq in the deeply sequenced modENCODE embryo samples [37] where it is known to be expressed in a few cells. This gene and others at similar expression levels missed by sequencing highlight the problem of detecting genes with low expression no matter what the technique, be it arrays or sequencing. This example aside, failure to obtain sufficient coverage and check the representation of this coverage (that is, library complexity) will provide erroneous metrics of gene expression and lead to false inferences even for genes that are detected. Given the current expense of RNA-Seq, and the excitement about the prospects of deep sequencing, this may cause some groups to avoid determining the coverage (that is, the number of reads) necessary to accurately sample the transcriptome of interest. High costs may also tempt some to avoid using biological replicates. These choices can lead to inaccurate estimates of gene expression level and thus false inferences [36]. Another source of bias in sequencing is the heterogeneity of reads across an expressed region - that is, uneven sequencing depth along the length of a transcript. This heterogeneity in coverage will influence expression estimates for transcripts and needs to be corrected [38-40]. Coverage and heterogeneity are not an issue in microarrays because of the fixed nature of probes that capture the transcripts by hybridization.

A final consideration about arrays and sequencing is the quantity and size of the data. In expression microarrays the raw data are composed of image files, typically TIFF files that may be around 30 MB per array. These TIFF files are transformed into text files that contain fluorescence intensities for each gene. The Illumina instrument generates upwards of 600 GB of data files but the sequence files (around 20-30 GB) are typically used as a starting point for analysis. These sequence files are an order of magnitude larger than those from arrays and because of these large file sizes, Python, Perl, Unix command line, and other scripting are necessary to sort and experiment with these files. Using spreadsheet software will not be an option and therefore bioinformatics support is necessary. For biologists unfamiliar with computer languages, there are growing alternatives for working with sequencing data. For example, many of the tools for sequencing data analysis are now available in Galaxy software, a web interface that provides a user friendly graphical interface [41,42].

## An example from the fruit fly

As a way to introduce and discuss microarrays and deep sequencing for measuring the transcriptome we will use a fly example from our own laboratory: specifically, experiments designed to profile gene expression in female and male heads of *Drosophila pseudoobscura*. This is one of several species of fly that we are profiling to validate evolutionarily novel *D. melanogaster *transcripts in the model organism Encyclopedia of DNA Elements (modENCODE) project [43]. We performed microarray and RNA-Seq experiments on the same samples and then compared expression measurements between microarrays and RNA-Seq.

**Figure 1:**
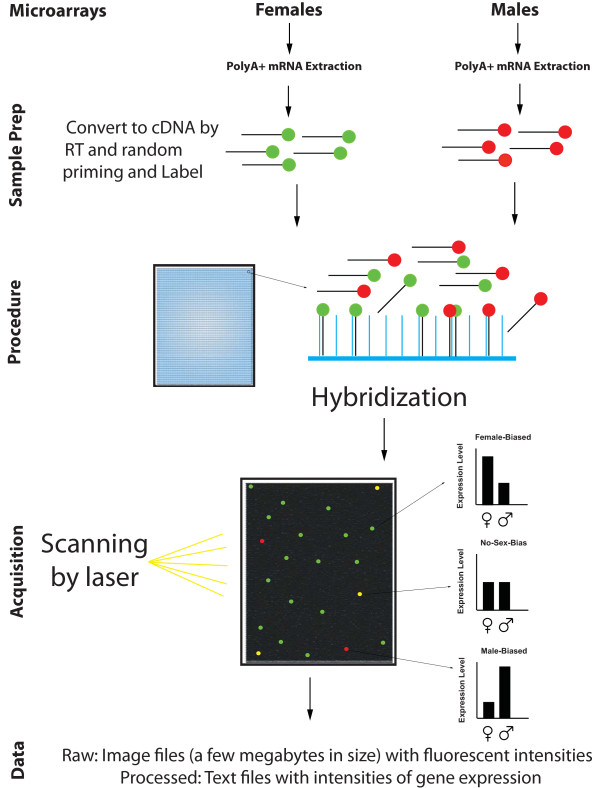
**Data production workflow for microarrays**. Microarrays require labeling of target material, hybridization to arrays, washing, and scanning to obtain measures of gene expression. RNA converted to cDNA from the sample will hybridize to the corresponding oligonucleotide targets, so that more highly expressed genes will be reflected in more abundant material hybridized and thus greater fluorescence intensity. In modern arrays, multiple probes are designed for a single gene in order to obtain fluorescence intensities that can be used as an index of gene expression.

Figure 1 describes an expression experiment designed to identify genes that are differentially expressed in *D. pseudoobscura *female and male heads, which were manually dissected from flies over dry ice, after which total RNA was extracted followed by a poly A+ selection. poly A+ selected mRNA was converted to cDNA using end-labeled random nonamers and reverse transcription. During this reaction, a fluorophore is added to the 5' end of each short cDNA. In this case, the cDNA of one sex was labeled with one type of fluorescent dye (cyanine 3 or Cy3) and the cDNA of the other sex was labeled with a different fluorescent dye (cyanine 5 or Cy5) with fluorescence at a different wavelength. We generated replicate samples (*N *= 4) and samples with dyes swapped between females and males in order to control for technical artifacts due to labeling and dye biases and to measure the inherent variability in gene expression irrespective of the sex of the sample.

As with any assay, replicate samples are critical for statistical analysis. The female and male labeled cDNA samples were mixed and applied to the microarray for hybridization. cDNAs that are complementary to probes on the microarray hybridize on the basis of simple first principles: more highly expressed genes will have more transcripts converted to labeled cDNA, and these more abundant cDNAs will bind more to their target probes than those of less expressed genes. Because we co-hybridized samples labeled with different fluorescent dyes we can take a ratiometric expression score between female and male heads: that is, genes that are more highly expressed in one sex than in the other will hybridize more to the target probe and generate a stronger signal. Genes that are expressed at the same level in both sexes will have equivalent amounts of transcript bound to probes and so the signal will be a combination of both Cy3 and Cy5 signal thereby generating a signal intermediate between the two (yellow fluorescence). The analysis and normalization methods for microarrays are highly developed [12] and thus this experiment should allow the differences in steady-state mRNA levels between female and male head tissue to be reliably measured.

**Figure 2:**
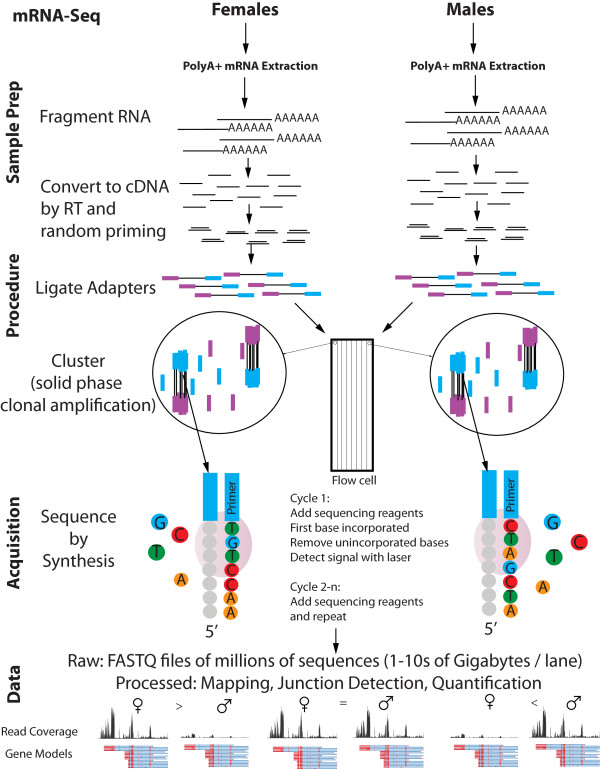
**Data production workflow for RNA-Seq**. RNA-Seq requires building libraries of fragmented RNA that are then converted to cDNA by reverse transcription, followed by adaptor ligation and size selection. Sequencing libraries are prepared for clustering on an 8 lane flow cell and sequencing-by-synthesis is used to generate tens of millions of sequences per sample that can be mapped to a reference genome. The number of reads that map to a scaled region of genome space are the index of the expression level of the gene.

Figure 2 shows the same analysis performed by RNA-Seq, using an Illumina Genome Analyzer and a commonly deployed protocol for preparing libraries [44]. First, the transcriptomes for females and males are fragmented by alkaline hydrolysis, then reverse-transcribed to make double-stranded cDNAs using random hexamer primers. Next, the ends of transcript fragments are prepared to enable oligonucleotide adaptors to be ligated onto the ends. Fragments are then size-selected, amplified by PCR and injected into a flow cell. The flow cell is a glass slide that contains a lawn of oligonucleotides complementary to the adaptors ligated to transcripts and with a series of separate lanes in which sequencing reactions take place.

Once the adaptors on the DNA fragments have hybridized to the complementary oligonucleotides in the flow cell, the fragments are amplified by isothermal bridge amplification to generate clusters of DNA clones. (In isothermal bridge amplification, the templates arch over and bind to adjacent oligonucleotides and then DNA polymerase copies the templates.) Double-stranded DNAs are denatured and the process is repeated to generate clusters of DNA clones. Next, the free 3' OH ends of the linearized clusters are blocked to prevent nonspecific sequencing reactions. Finally, the clusters are denatured and a sequencing primer is hybridized to the linearized and blocked clusters.

Sequencing reactions consist of a series of reactions to image individual bases within each cluster. Bases are imaged by using reversible fluorophore terminator nucleotides. The first base in the cluster is identified by adding four labeled reversible terminators, primers, and polymerase. A laser is used to excite the fluorophores and this allows identification of the first base. The next cycle repeats the incorporation of four reversible terminator nucleotides, primers, and polymerase. A laser again excites the terminators and bases are identified. These cycles of adding reagents, followed by laser excitation, and data capture are repeated to produce a read and typical reads range from 25 to over 75 base pairs in size. At the end of a run (3-7 days or more depending on read length) there are 30-40 million (possibly more) high quality sequences.

The RNA-Seq measure of gene expression is density of reads mapping to a particular transcript. For species with sequenced genomes, a common method is to map reads to a reference genome. Illumina provides a mapper called ELAND but many free open source tools are available. The tools that we have used most extensively for RNA-Seq are the Tuxedo Suite Tools (Bowtie [45], a short read mapper; Tophat [46], a splice junction identifier, and Cufflinks [33], a transcript assembler). Two expression metrics are commonly used which provide a value normalized by overall sequencing depth, FPKM (expected fragments per kilobase of transcript per million fragments mapped) and RPKM (reads per kilobase per million mapped reads) [23,33,40], which are conceptually similar. In the example given in Figure 2, we estimate expression in units of RPKM by quantifying reads that map with genes predicted from genomic sequence. Therefore, higher RPKM in females would be examples of genes with female-biased expression, higher RPKM for males would be genes with male-biased expression, and equivalent RPKM in both sexes would be examples of non-sex-biased genes.

## Do arrays and RNA-Seq tell a consistent story?

**Figure 3:**
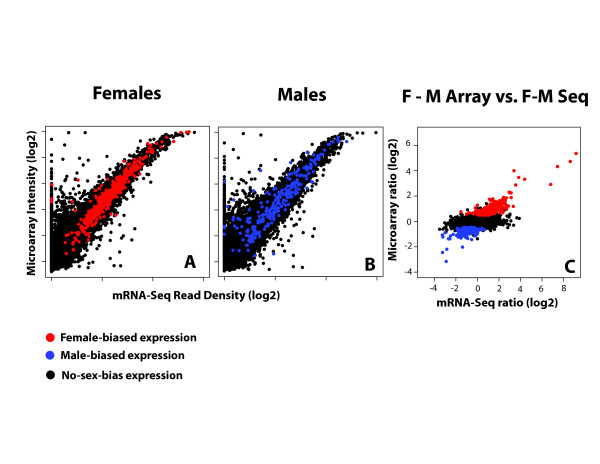
**Comparison of array and RNA-Seq data for measuring differential gene expression in the heads of male and female *D. pseudoobscura***. **(a) **Results for female heads; **(b) **results for male heads. We used custom designed Nimblegen arrays to an early release of the *D. pseudoobscura *annotation. This array consists of 50-mer probes selected without bias to gene position, and with an average of 10 probes per gene model. A full description of this array platform can be found in the GEO under platform number GPL4631. Robust Multi-array Averaging (RMA) [50] was used to normalize array experiments and normalization improves the correlation between arrays and sequencing results. A full description of the analysis and all sequencing data can be found in [51]. Colored circles are genes identified as differentially expressed between females and males by microarray analysis with four biological replicates. In this case, one of the four biological replicates was prepared for sequencing by fragmenting RNA using alkaline hydrolysis and constructing a cDNA library for sequencing. For these analyses, we generated about 6 million 36 base pair reads from the Illumina GA I platform and the number of reads per kilobase per million mapped reads (RPKM) was calculated by counting the number of unique mapping reads from the default Illumina mapper (ELAND but the same pattern holds for Bowtie) to the same coding sequence models that were used for constructing probes for the microarray. The correlation between fluorescence intensity as a surrogate for gene expression and the RPKM metric as obtained by mRNA-Seq is high (Pearson's *r *= 0.90-0.91; Spearman's *rho *= 0.90-0.91) and slightly higher for just the genes identified as differentially expressed by microarrays (Pearson's *r *= 0.89-0.92; Spearman's *rho *= 0.90-0.94). In the case of fold change **(c) **measurements (female/male), the congruence is reasonable for the entire data set (Pearson's *r *= 0.62; Spearman's *rho *= 0.54) but high in the case of the fold change measurements for the genes with sex-biased expression (Pearson's *r *= 0.92; Spearman's *rho *= 0.89).

A key first question is whether, when used to ask exactly the same question, both techniques give the same answer. Comparing expression metrics from array intensities to RNA-Seq density shows a strong congruence (Figure 3). The relationship is not quite linear, as there appears to be a slight compression in the array data at the high end, but the vast majority of the expression values are similar between the methods. Scatter increases at low expression, which is not surprising, as background correction methods for arrays are complicated when signal levels approach noise levels. Similarly, RNA-Seq is a sampling method and stochastic events become a source of error in the quantification of rare transcripts [47]. There is, however, one consistent difference in our comparisons in *Drosophila*. There is a large range of expression values at the low end on arrays that that are undetectable by RNA-Seq. We cannot explain this difference, but whatever the cause, it does not affect the measurement of differential expression at expression levels that are detectable by RNA-Seq (Figure 3). In our experiment, we used biological replicate samples for the arrays and applied moderated *t*-tests to detect those genes that were differentially expressed between females and males. In the analysis in Figure 3, our goal was to compare expression measurements between the platforms. The genes showing sex-biased expression (red and blue dots in Figure 3) are in outstanding agreement between microarrays and RNA-Seq. We have observed similar congruence in the extremely deep RNA-Seq data in modENCODE *D. melanogaster *female and male samples [37]. Annotated sex-biased genes based on the extensive array-based literature [48] and the deeply sequenced modENCODE samples report the same biology.

## The answer is yes

Both sequencing and hybridizing mRNA to arrays are high-throughput ways to profile the transcriptome and for problems that can be addressed by both, they show similar performance and complement each other [29,47,49]. Detecting genes with low expression will remain a problem for both techniques, but there are some applications, such as transcript discovery and isoform identification, where RNA-Seq will be the better choice. Given the substantial agreement between the two methods, the array data in the literature should be durable.
